# Comparative ecotoxicological study on the current status of artisanal crude oil contaminated mangrove swamps in Rivers State, Southern Nigeria

**DOI:** 10.1016/j.heliyon.2024.e34588

**Published:** 2024-07-14

**Authors:** Davies Ibienebo Chris, Nwosu Obiageli Juliana, Okechukwu Kenneth Wokeh, Azra Mohamad Nor, Fathurrahman Lananan, Lee Seong Wei

**Affiliations:** a Department of Fisheries, University of Port Harcourt, Port Harcourt, East-West Road, Choba, Rivers State, P.M.B. 5323, Nigeria; b Department of Animal and Environmental Biology, Faculty of Science, University of Port Harcourt, P.M.B 5323, Choba, Rivers State, Nigeria; c Institute of Climate Adaptation and Marine Biotechnology, Universiti Malaysia Terengganu (UMT), 21030, Kuala Nerus, Terengganu, Malaysia; d Research Center for Marine and Land Bioindustry (Earth Sciences and Maritime), National Research and Innovation Agency (BRIN), Pemenang, West Nusa Tenggara, 83352, Indonesia; e East Coast Environmental Research Institute, Universiti Sultan Zainal Abidin (UniSZA), Gong Badak Campus, Kuala Nerus, 21300, Terengganu, Malaysia; f Department of Agricultural Sciences, Faculty of Agro-Based Industry, Universiti Malaysia Kelantan, Jeli Campus, 17600, Jeli, Kelantan, Malaysia

**Keywords:** Physicochemical parameters, Public health, Mudskipper (*Periophthalmus papillio*)

## Abstract

The rise in illegal crude oil theft and refining in the southern Niger Delta region of Nigeria, especially in Rivers State, has led to significant environmental damage to aquatic ecosystems. A study was carried out to assess the impact of crude oil bunkering on aquatic environments and fish samples from Oproama, Sama-Naguakiri, and Abalama over six months. Findings revealed that Oproama had the highest levels of biological oxygen demand (3.60 ± 0.79 mg/L), electrical conductivity (34.07 ± 3.62 μS/cm), total dissolved solids (28.17 ± 3.77 mg/L), and temperature (29.50 ± 0.74 °C). In contrast, Sama-Naguakiri recorded the highest pH (6.72 ± 0.14) and dissolved oxygen (3.35 ± 0.11 mg/L). Though minor variances were noted between Sama-Naguakiri and Abalama, a significant difference (P < 0.05) was observed between these areas and Oproama. Importantly, all measured values adhered to WHO/FAO standards. Analysis of potentially harmful metals in sediment and water indicated notable distinctions among the three sites, with Sama-Naguakiri exhibiting the highest levels of Zn (114.5 ± 1.5 mg/kg), Cu (237.8 ± 0.9 mg/kg), Pb (3.6 ± 1.2 mg/kg), and Cd (1.1 ± 0.4 mg/kg). Conversely, Abalama showed the lowest zinc (105.2 ± 1.5 mg/kg) and lead (2.4 ± 0.5 mg/kg) concentrations, while Oproama displayed the lowest copper level (0.8 ± 0.3 mg/kg). The concentrations of heavy metals in the water, sediment, and fish surpassed the permissible limits established by NESREA, the EPA, and WHO, except for arsenic. The presence of heavy metals in this region could pose significant ecological and health hazards, underscoring the urgency for immediate remedial measures to safeguard the environment and this fish-dependent community.

## 1 Introduction

Coastal rivers and estuaries, acknowledged as pivotal ecosystems on a global scale, confront escalating threats attributed predominantly to human activities, notably the expansive landscape of oil production [1,2]. These dynamic environments exhibit spatial and temporal fluctuations, influenced by multifaceted biogeochemical forcings such as sediment composition, pH, temperature, and anthropogenic perturbations [3,4]. According to Landrigan et al. [5]. the intricate nature of these ecosystems makes them particularly vulnerable to disruptions, with oil pollution emerging as a primary catalyst for adverse impacts.

Oil pollution within coastal rivers manifests through oxygen deficiency, toxicity, and coating, jeopardizing fundamental life-support processes and thereby posing threats to community resilience and population dynamics [6,7]. The intricate interplay of factors, including water and sediment quality, becomes paramount in understanding and mitigating the ecological repercussions of anthropogenic disturbances.

Sediments, recognized as crucial components within river ecosystems, function as contamination sinks [[8], [9], [10]]. Their physicochemical properties and response to hydrological dynamics play a pivotal role in mediating contamination to linked ecosystem components [11]. Significant biota, water and sediment contamination can trigger biodiversity losses and initiate deleterious food chain reactions, affecting both aquatic life and human well-being [7,12,13].

The inherent connection between sediment chemistry and the observed biological impacts on organisms continues to pose a challenge in environmental science. Screening guidelines, indices, or benchmarks have been suggested to assess the likelihood of biological effects based on chemical levels in sediments [11,14]. This highlights the importance of analyzing biota, water, and sediment quality in monitoring and maintaining ecosystem health, even if the overlying water meets established quality standards.

Aquatic systems, comprising biota, surface water and sediment, stand as highly contaminated environments globally [15]. The pathways through which pollutants, including heavy metals, infiltrate these systems are diverse, ranging from atmospheric deposition to surface water runoffs and the leaching of contaminants from terrestrial sources [16]. This influx of pollutants places significant strain on aquatic ecosystems and underscores the importance of effective monitoring and regulatory measures.

Fish serve as a valuable source of protein, essential minerals, vitamins, and unsaturated fatty acids, contributing significantly to human nutrition [17,18]. However, the presence of heavy metals in fish can compromise these health benefits due to their tendency to accumulate and magnify within fish tissues, a phenomenon known as bioaccumulation and biomagnification [10]. Heavy metals are notorious for their capacity to inflict adverse health effects on humans, encompassing conditions such as liver damage, renal failure, cardiovascular diseases, and even mortality [19].

Preserving the quality of aquatic ecosystems below a critical threshold has become an imperative goal. Regrettably, in recent years, this goal has become increasingly challenging due to the runoff of contaminants, atmospheric deposition, and uncontrolled discharge of effluents from urban, municipal, and industrial sources into streams, rivers, and marine waters [20]. Furthermore, domestic and industrial waste is routinely deposited into water bodies, a problem that is particularly pronounced in Africa where the enforcement of environmental protection laws is often lax. Consequently, heavy metals and other hazardous substances within these wastes accumulate in bottom sediments.

As a result, coastal communities are grappling with the direct consequences of toxic substances in water and sediments, as well as the transfer of these contaminants through aquatic food chains [21]. Previous studies have underscored the harmful effects of prominent environmental pollutants like Arsenic (As), Cadmium (Cd), Copper (Cu), Lead (Pb), Zinc (Zn), and Iron (Fe) on aquatic ecosystems [[22], [23], [24]]. Some coastal communities in Rivers State, Southern Nigeria, are particularly vulnerable to artisanal crude oil refining and other anthropogenic activities with detrimental consequences. Notably, communities like Oproama, Sama-Naguakiri, and Abalama, all located in the Asari-Toru area of the Niger Delta, face these environmental challenges.

These communities located along the Sombrero River are particularly vulnerable to pollution pressures from crude oil contaminants, waste inputs, and urban and industrial growth. Despite being less studied than estuaries like Bonny and New Calabar, these creeks demand attention for remediation and monitoring. This research aims to evaluate the existing condition of crude oil-contaminated mangrove swamps in southern Nigeria. This assessment will encompass an investigation into potentially toxic elements present in fish, water, and sediment samples.

The nexus between environmental degradation, human activities, and ecological consequences is particularly pronounced in these mangrove swamps. Addressing these challenges necessitates a multi-faceted approach, encompassing monitoring, assessment, and the implementation of robust remediation strategies. Collaborative research endeavors, guided by stringent environmental regulations, become imperative for safeguarding the health of these ecosystems and ensuring the well-being of coastal communities in the Niger Delta region.

The intricacies of coastal river and estuarine ecosystems demand nuanced understanding and concerted efforts to mitigate the impacts of anthropogenic activities. The preservation of these vital ecosystems relies on interdisciplinary research, robust regulatory frameworks, and collaborative initiatives to ensure sustainable and resilient aquatic environments for current and future generations.

## 2 Materials and methods

### 2.1 Study area

**Fig. 1:**
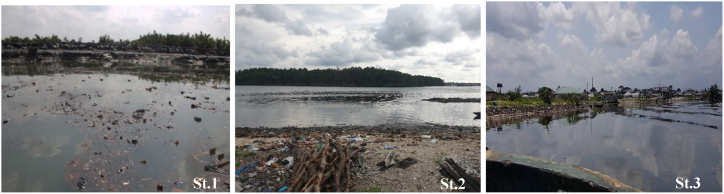
Showing the Sama-Naguakiri (St.1), Oproama and (St.2), and Abalama (St.3) stations.

**Fig. 2:**
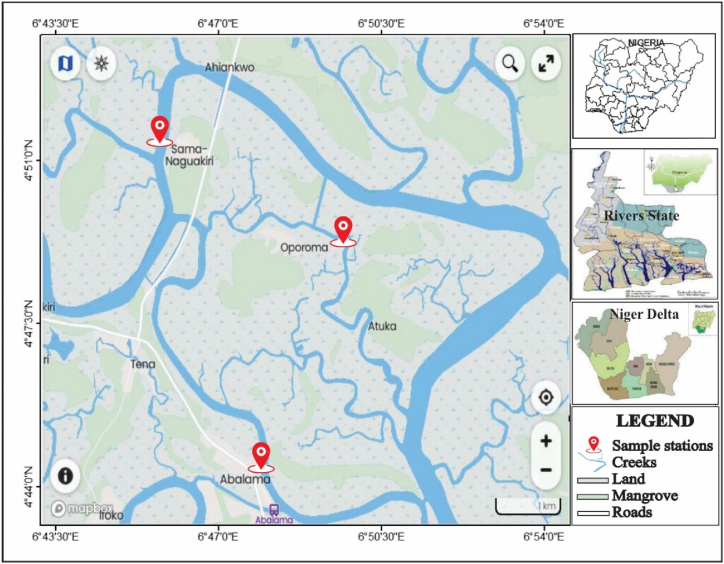
Map showing the study area.

The study was conducted in the Asari-Toru Local Government Area of Rivers State, Nigeria, focusing on three communities: Opuro-ama, Sama-Naguakiri, and Abalama. These communities are located near the Sombrero Estuary, a part of the Niger Delta coastal region. The local ecosystem is characterized by soft intertidal bottom habitats, a diverse range of fish families, and mangrove vegetation. The sediment in the area is characterized by spongy, fibrous "chicoco" peaty mud. Despite being one of the few remaining intact mangrove belts in the eastern Niger Delta, these mangroves face threats from illegal refining, bunkering, sand dredging, and deliberate mangrove cutting. The research used a quantitative approach and strategically placed approximately 1000 m apart to ensure that a broader area including sites with various contaminants from visible illegal refining waste effluents, was adequately represented (as depicted in Fig. 1, Fig. 2).

### 2.2 Sample and sampling procedure

In order to represent various activities in catchments and their importance as pollution sites, three sampling stations were chosen in each of the communities based on the peculiarities and features observed around the study area of the creeks. The sampling stations were selected to reflect different activities in the areas at least 1000 m apart along each of the creeks. Heavy metals of interest which are Lead (Pb), Copper (Cu), Iron (Fe), Cadmium (Cd), Zinc (Zn), and Arsenic (As) were chosen on the suspicion that they have a high prevalence in the study area giving the nature of industrial and domestic effluents traversing the study area. Detailed descriptions of sampling points and the corresponding activities in the vicinity are given in Table 1. Samples collected for analysis include sediment, water and biota (mudskipper). All sites were geo-referenced using a handheld global positioning system (GPS) receiver unit (Magellan GPS 315) to generate geographic coordinates (longitudes and latitudes) of the sampling area. Sampling was carried out ones in a month between October 2021 to April 2022.

**Table 1:** Description of sampling points.

Locations	Stations	Latitude	Longitude	Surrounding Activities
Sama-Naguakiri	**1**	E007^°^30′55	N04^°^49′41.0”	Illegal refining waste effluent site within view, refuse and human waste disposal, dredging and fishing.
Oproama	**2**	E006^°^50′16	N04^°^48′14.0”	Domestic wastes, evident runoffs track of illegal refining waste effluent, refuse and waste disposal, dredging and fishing.
Abalama	**3**	E006^°^50′25	N04^°^45′47”	Illegal refining waste effluent sites not within view, refuse and human waste disposal, dredged sites and fishing activities.

### 2.3 Collection of samples and analysis

The collection of interstitial water samples was conducted using high-density Schott glass vials that had undergone a meticulous cleaning procedure. The bottles went through a pre-cleaning process involving the use of detergent and subsequent rinsing with tap water. Following that, the specimens were subjected to a 24-h immersion in a hydrochloric acid (HCl) solution with a concentration of 50 %. After undergoing acid treatment, the bottles were meticulously cleansed using tap water and subsequently rinsed extensively with triple distilled water. The implementation of a meticulous cleaning protocol was important in order to mitigate the risk of possible contamination caused by metal residue and to deter the adhesion of metals onto the glass surface.

After the collection of samples, they were swiftly packed and brought to the laboratory, ensuring their integrity was maintained by storing them in an ice pack. The gathered samples were meticulously labelled and securely stored in refrigeration prior to undergoing examination. The analysis procedure adhered to the established and well-recognized standard methodologies as suggested by the American Public Health Association (APHA) (2005) for the quantification of heavy metal concentrations in the samples.

Moreover, a number of crucial physicochemical properties were directly evaluated at the locations where the samples were taken. The factors used in this study were Dissolved Oxygen (DO), temperature, hydrogen ion concentration (pH), conductivity, and Total Dissolved Solid (TDS). In order to determine the pH level, a compact pH metre, namely the Milwaukee model pH600, was utilized. Dissolved oxygen levels were measured via a dissolved oxygen meter, namely the MW 600 Model. Furthermore, the levels of Total Dissolved Solids (TDS) in parts per million (ppm), conductivity, and temperature were accurately assessed by utilising a portable multi-meter (EZODO Multi-meter model CTS-406).

In addition, the laboratory conducted an assessment of Biochemical Oxygen Demand (BOD) using the 5-day BOD test, following the guidelines established by APHA [25]. The use of this thorough methodology for data gathering and analysis guarantees the dependability and precision of the acquired outcomes.

The study involved the collection of mudskipper fish (*Periophthalmus papillio*) from three distinct sampling stations within each community. This collection procedure took place at regular intervals of twenty-one days over a span of six months, specifically from October 2021 to March 2022. As a result, a total of sixty representative fish samples were obtained, with an average length of 9.5 ± 0.65 cm and weight of 10.3 ± 0.87g. Each community yielded fifteen samples per station. *P. papillio* specimens were also collected, as they exhibit a strong association with mangroves and the surrounding habitats. The laboratory conducted an analysis of the fish's heavy metal content using the standard analytical procedures outlined in APHA [25]. Sediment samples of a composite nature were gathered on a monthly basis over a span of six months from three distinct sites, employing an "Ekman grab" sampler. The specimens were placed in plastic receptacles that had been subjected to a 10 % nitric acid treatment for a duration of 24 h, followed by a thorough rinsing with de-ionized water. The specimens were conveyed to the laboratory and maintained at a frozen state of −20 °C till further examination utilising the Atomic Absorption Spectrophotometric Machine (API-RP 45).

### 2.4 Quality assurance and control

The experimental setup utilized NIST-certified atomic absorption standards to establish a calibration curve for various heavy metals. To ensure measurement reproducibility, a reagent blank was performed following every 10 samples to mitigate equipment drift. Recovery rates varied from 82 to 110 percent. Metal concentrations in water, soil, and biota samples were assessed through atomic absorption spectrophotometry, specifically employing Buck Scientific's Model 210VGP. Chris et al. [10] outlined the methodology and wavelength (nm). All samples underwent analysis in triplicate, and the reported findings reflect the average of these measurements. This meticulous approach to quality assurance and control enhances the accuracy and dependability of the results, as all experimental procedures utilize analytical-grade chemicals.

### 2.5 Determination of heavy metals

The study employed the Atomic Absorption Spectrophotometer (AAS) to determine the levels of Lead (Pb), Copper (Cu), Iron (Fe), Cadmium (Cd), Zinc (Zn), and Arsenic (As) in the water samples. The procedure outlined by APHA [25] was meticulously followed, ensuring strict adherence to the manufacturer's recommendations for configuring the instrument settings and operational parameters.

### 2.6 Total digestion for heavy metals during analysis

The sediment samples were subjected to an air-drying procedure, while the fish samples were pulverised using a blender. The water samples were treated with 20 ml of pure H2SO4 for digestion. The heavy metal analysis methodology adhered to the requirements stipulated by the American Public Health Association (APHA). The extraction procedure utilized a modified methodology using Nitric acid, Perchloric Acid, and Sulphuric acid (APHA 3030I modified - full digesting method).The experimental protocol started with the measurement of 1 g of silt or granulated fish samples, which was subsequently transferred into a 250 ml Pyrex conical flask. Following that, a volume of 20 ml of the digestion solution was introduced to the samples. Subsequently, the mixes underwent heating on an electric mantle at a temperature of 250 °C till the granules underwent a transition from a black hue to a grey shade, assuring the thorough digestion of the samples. Following the application of heat, the solution that had undergone digestion was removed from the fume hood and subsequently allowed to cool. Subsequently, a volume of 20 ml of distilled water was added. The solution that had undergone digestion was then passed through a Whatman 42 ashless filter paper and collected in a 100 ml glass volumetric flask. Subsequently, the filtrate that had undergone digestion was carefully adjusted to the 100 ml mark on the volumetric flask. It was then transferred to a new 100 ml plastic bottle, which was appropriately labelled and prepared for the measurement of Lead (Pb), Copper (Cu), Iron (Fe), Cadmium (Cd), Zinc (Zn), and Arsenic (As) using Atomic Absorption Spectroscopy (AAS).

### 2.7 Evaluation of tissue concentration

The Bioaccumulation Factor (BAF) was computed to assess the extent of heavy metal buildup in fish tissue. This calculation was performed using the following formula:

**BAF** = (Concentration of Heavy Metal in Fish Tissue)/(Concentration of Heavy Metal in Sediment or Water)

This formula determined the concentration of heavy metals in fish tissue concerning the sediment or water (mg/kg or ml/L).

### 2.8 Statistical analysis

The data were subjected to statistical analysis using SPSS version 16, which involved descriptive statistics and analysis of variance (ANOVA). The Duncan Multiple Range Test, with a significance level of 0.05, was employed to distinguish significant means. Fish tissue concentrations were determined using the mean values generated. Microsoft Excel software was utilized for data interpretation, with the statistical parameters utilized being the mean, standard deviation, and standard error of the mean. Pearson's Product Correlation was applied to investigate the associations among the analysed variables.

## 3 Results

### 3.1 Spatial variation in physicochemical parameters across stations

Spatial variations in physicochemical parameters across the stations are presented in Table 2. The temperature, pH, dissolved oxygen (DO), salinity, biochemical oxygen demand (BOD), and total dissolved solids (TDS) values exhibited significant differences (P < 0.05) between Oproama and the other two stations, Abalama and Sama-Naguakiri. The temperature was significantly higher in Oproama (29.50 ± 0.74 °C) and lower in Sama-Naguakiri (25.75 ± 0.57 °C) while the pH value was higher at Sama-Naguakiri (6.72 ± 0.14) and least in Oproama (5.68 ± 0.18). The highest dissolved oxygen (DO) concentration was observed at Sama-Naguakiri (3.35 ± 0.11 mg/L), followed by Abalama (3.27 ± 0.21 mg/L), with Oproama having the lowest DO concentration (2.92 ± 0.20 mg/L). Salinity was highest at Oproama (15.28 ± 0.71 ppm), followed by Abalama (12.78 ± 0.81 ppm), and Sama-Naguakiri had the lowest salinity (11.44 ± 0.45 ppm). The highest biochemical oxygen demand (BOD) value was recorded at Oproama (3.60 ± 0.79 mg/L), while the lowest BOD value was observed at Sama-Naguakiri (2.46 ± 0.42 mg/L). Electrical conductivity values were highest at Oproama (34.07 ± 3.62 μS/cm), followed by Abalama (28.78 ± 2.610 μS/cm), and the lowest electrical conductivity value (25.76 ± 0.47 μS/cm) was found at Sama-Naguakiri. Total dissolved solids (TDS) were highest at Oproama (28.17 ± 3.77 mg/L), while the lowest TDS value was recorded at Sama-Naguakiri (19.20 ± 2.29 mg/L). Significantly, there were no differences (P > 0.05) observed in these parameters between the Sama-Naguakiri and Abalama sampling stations.

**Table 2:** Mean spatial variability in the physicochemical parameters across the stations.

Stations	Temperature (^o^C)	pH	DO (mg/L)	Salinity (ppm)	BOD (mg/L)	Cond (μS/cm)	TDS (mg/L)
[26]	30	6.6–8.5	6	120	10	600	500
Sama-Naguakiri	25.75 ± 0.57^b^	6.72 ± 0.14^b^	3.35 ± 0.11^a^	11.44 ± 0.45^b^	2.46 ± 0.42^b^	25.76 ± 0.47^b^	19.20 ± 2.29^b^
Oproama	29.50 ± 0.74^a^	5.68 ± 0.18^a^	2.92 ± 0.20^b^	15.28 ± 0.71^a^	3.60 ± 0.79^a^	34.07 ± 3.62^a^	28.17 ± 3.77^a^
Abalama	27.40 ± 0.75^ab^	6.45 ± 0.18^b^	3.27 ± 0.21^a^	12.78 ± 0.81^b^	2.78 ± 0.47^b^	28.78 ± 2.61^b^	21.95 ± 2.50^b^

WHO: World Health Organization. The results were presented as mean ± standard deviation of triplicate determinations. Means in the same column with different superscripts (a, b, c) are considered significantly different (P < 0.05).

WHO: World Health Organization. The results were presented as mean ± standard deviation of triplicate determinations. Means in the same column with different superscripts (a, b, c) are considered significantly different (P < 0.05).

### 3.2 Temporal variations in physico-chemical parameters

Table 3 shows the temporal variation in physicochemical parameters (pH, temperature, salinity, BOD, electrical conductivity, TDS and DO) from October 2021 to March 2022. The temperature ranged between 25.60 ± 0.87 °C to 29.50 ± 1.46 °C and mean pH levels ranged between 5.80 ± 0.38 to 6.77 ± 0.26. The DO ranges between 2.57 ± 0.33 mg/L to 3.53 ± 0.18 mg/L. Salinity ranged between 11.63 ± 1.00 to 15.57 ± 1.57 and mean electrical conductivity ranged between 23.85 ± 1.88 to 38.63 ± 6.39. The BOD ranged between 2.43 ± 0.09 to 4.80 ± 0.09 while the TDS ranged between 17.20 ± 0.81 to 36.37 ± 4.55 mg/L. The temperature was lowest in October, pH was lowest in February and DO was in January. Salinity and BOD were lowest in October and the electrical conductivity was lowest in March, while TDS was in December. Temperature and electrical conductivity and TDS were highest in February, pH in March, DO was in December while salinity was in January and BOD was highest in March. The monthly variation in temperature, salinity BOD, electrical conductivity, TDS and DO was significantly different (P > 0.05) with variation between some months. However, there was no statistically significant fluctuation (P > 0.05) observed in pH levels across all the months.

**Table 3:** Temporal variation in physico-chemical parameters.

Months	Temperature (^o^C)	pH	DO (mg/L)	Salinity (ppm)	BOD (mg/L)	Cond (μS/cm)	TDS (mg/L)
[[26], [27]]	30	6.6–8.5	6	120	10	600	500
October	25.60 ± 0.87^c^	6.40 ± 0.30^a^	3.33 ± 0.22^a^	11.63 ± 1.00^b^	2.43 ± 0.09^b^	27.57 ± 0.97^b^	18.80 ± 1.91^c^
November	25.80 ± 1.18^c^	6.19 ± 0.18^a^	3.53 ± 0.18^a^	11.97 ± 1.05^b^	2.57 ± 0.27^b^	26.50 ± 0.40^b^	21.20 ± 3.72^b^
December	27.07 ± 1.08^b^	6.47 ± 0.40^a^	3.37 ± 0.12^a^	13.33 ± 1.14^ab^	3.10 ± 0.60^ab^	27.83 ± 2.23^b^	17.20 ± 0.81^c^
January	28.33 ± 1.07^b^	6.07 ± 0.49^a^	2.57 ± 0.33^b^	15.57 ± 1.57^a^	4.10 ± 0.62^a^	32.83 ± 3.84^a^	22.50 ± 3.11^b^
February	29.50 ± 1.46^a^	5.80 ± 0.38^b^	3.23 ± 0.30^a^	13.97 ± 1.37^a^	4.69 ± 0.60^a^	38.63 ± 6.39^a^	36.37 ± 4.55^a^
March	29.00 ± 0.94^a^	6.77 ± 0.26^a^	3.03 ± 0.09^a^	12.56 ± 1.18^b^	4.80 ± 0.09^a^	23.85 ± 1.88^c^	22.58 ± 2.49^b^

WHO: World Health Organization.

The results were presented as mean ± standard deviation of triplicate determinations.

Means in the same column with different superscripts (a, b, c) are considered significantly different (P < 0.05)

### 3.3 Correlation among physicochemical parameters in the study area

Table 4 presents the correlation analysis of physicochemical characteristics within the designated research region. A significant positive association was found between temperature and salinity (r = 0.630), dissolved oxygen (r = 0.246), conductivity (r = 0.501), and total dissolved solids (r = 0.695). A positive association was found between pH and dissolved oxygen (DO) with a coefficient of 0.092. Conversely, pH showed a negative correlation with salinity (−0.500). Additionally, DO exhibited strong negative correlations with salinity (−0.917), conductivity (−0.901), and total dissolved solids (TDS) (−0.688). All the correlations between dissolved oxygen and other metrics exhibited a negative relationship. There was a positive association seen between salinity and dissolved oxygen (0.675), conductivity (0.645), and total dissolved solids (0.350). Significant and strong positive relationships were identified between biochemical oxygen demand (BOD) and electrical conductivity (r = 0.924), which in turn exhibited a positive association with total dissolved solids (TDS) (r = 0.546). A significant positive association was established between the electrical conductivity and total dissolved solids (TDS) with a correlation coefficient of 0.802.

**Table 4:** Correlation between physicochemical parameters in the study area.

	Tempt	pH	DO	Salinity	BOD	Conduct	TDS
Temperature (^o^C)	1						
PH	−0.199	1					
DO (mg/l)	−0.567	0.092	1				
Salinity (ppm)	0.630	−0.500	−0.789	1			
BOD (mg/l)	0.246	**−0.917**^b^	−0.203	0.675	1		
Conductivity (μS/cm)	0.501	**−0.901**^b^	−0.263	0.645	0**.924**^a^	1	
TDS (ppt)	0.695	−0.688	−0.114	0.350	0.546	0.802	1

a Correlation is significant at the 0.01 level (2-tailed).

b Correlation is significant at the 0.05 level (2-tailed).

### 3.4 Heavy metal concentrations across the three studied mediums

**Fig. 3:**
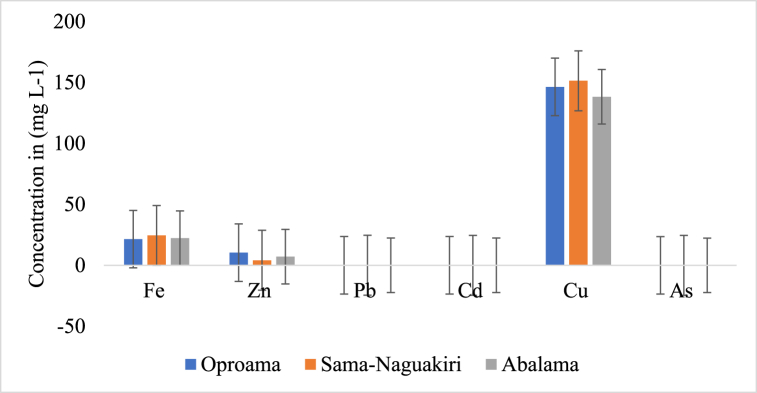
Display the average heavy metal concentrations in the water at each station.

**Fig. 4:**
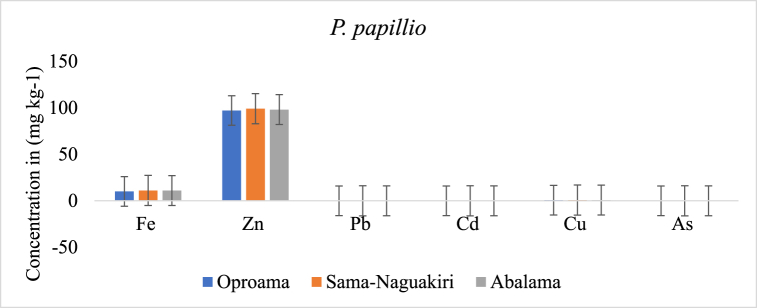
Present the average concentration of heavy metals in *P. papillio* for each station.

**Fig. 5:**
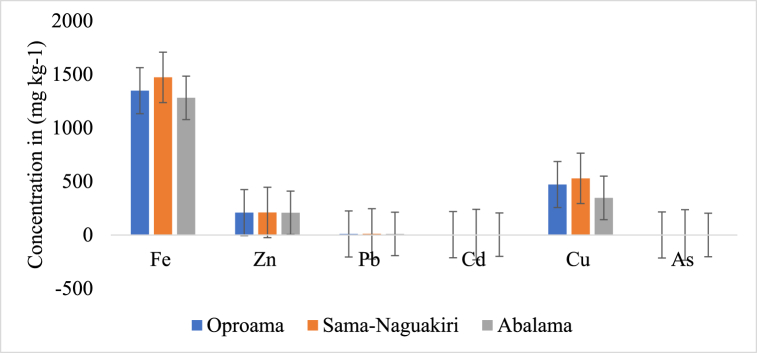
Display the average heavy metal concentrations in the sediment for each station.

Fig. 3, Fig. 4, Fig. 5 represent the average amount of heavy metals across the three media. The silt in Sama-Naguakiri exhibited the greatest Fe concentration, measuring at 656.6 ± 2.2 mg/kg. In comparison, the Fe concentration in the water was recorded at 36.69 mg/L, while the lowest Fe concentration was seen in the fish, measuring at 11.77 mg/kg. Furthermore, the sediment exhibited the greatest concentration of Zn, with a mean value of 217.6 ± 2.44 mg/kg. This was followed by the fish samples, which had a mean concentration of 103.2 ± 2.93 mg/kg. The lowest concentration of Zn was found in the water samples, with a mean value of 10.6 ± 1.40 mg/L. These measurements were obtained from the Sama-Naguakiri location. The concentration of Pb was found to be the greatest in sediment (8.44 ± 0.27 mg/kg) and the lowest in fish (0.01 ± 0.01 mg/kg). The sediment exhibited the greatest quantity of Cd at 2.7 ± 0.12 mg/kg, whereas the lowest value was seen in fish at 0.01 ± 0.01 mg/L. At the Sama-Naguakiri site, the sediment exhibited the greatest reported concentration of Cu (506.2 ± 2.77 mg/kg), followed by water (147.3 ± 6.02 mg/L), while the lowest concentration was seen in fish (0.7 ± 3.45 mg/kg).

### 3.5 The pattern of heavy metal concentrations throughout the study area

The concentration of heavy metals at each station followed this order of magnitude: station I (Sama-Naguakiri) > station II (Oproama) > station III (Abalama), as depicted in Fig. 3, Fig. 4, Fig. 5. The distribution sequence of heavy metals in water, fish, and sediment is as follows: Water = Cu > Fe > Zn > Pb > Cd > As; Fish = Zn > Fe > Cu > Pb=As=Cd; Sediment = Fe > Cu > Zn > Pb > Cd > As. Nonetheless, the sequence of heavy metal distribution in the three stations (Oproama, Sama-Naguakiri, and Abalama) was as follows: Oproama = Fe > Cu > Zn > Pb > Cd > As; Sama-Naguakiri = Fe > Cu > Zn > Pb > Cd > As; Abalama = Fe > Cu > Zn > Pb > Cd > As). No significant difference (P > 0.05) was observed in the As values in the sediment, fish, and water. The concentrations vary widely between stations and for each of the heavy metals in the fish (*P. papillio*) tissue. However, the sequence of heavy metal distribution in the fish tissue at each station followed the same trend: Oproama (Fish = Zn > Fe > Cu > Pb=As=Cd); Abalama (Fish = Zn > Fe > Cu > Pb=As=Cd); Sama-Naguakiri (Fish = Zn > Fe > Cu > Pb=As=Cd). Accumulation patterns remained consistent throughout all the months and stations, with the accumulation rates of all metals.

### 3.6 Biota-water accumulation factor (BWAF) of heavy metals in water within *P. papillio* tissue

Table 5 shows the variations in heavy metal concentrations in *P. papillio* tissue throughout three different stations (Oproama, Abalama, and Sama-Naguakiri). The tissue of P. papillio showed a range of Fe concentrations, relative to water, from 0.312 to 0.326 mg/kg, with the highest levels seen. The concentration range of Zn was seen to be between 9.517 and 10.050 mg/kg, whilst Pb exhibited variability within the range of 0.083–0.144 mg/kg, and Cd had a concentration range spanning from 0.167 to 0.25 mg/kg. The concentration of arsenic (As) remained consistent at 1 mg/kg across all instances. The findings suggest that there are notable variations in the levels of heavy metals in the tissues of *P. papillio* throughout the three designated locations. The tissue concentration sequence, arranged in descending order, for each station is as follows: In the Sama-Naguakiri community, the order of preference for metals is as follows: Zinc, Arsenic, Iron, Cadmium, Lead, and Copper. Similarly, in the Oproama community, the preferred order is Zinc, Arsenic, Iron, Cadmium, Lead, and Copper. Likewise, in the Abalama community, the preferred order is Zinc, Arsenic, Iron, Cadmium, Lead, and Copper. Regarding the element Fe, the tissue concentration exhibited the maximum value in Oproama (0.326 mg/kg), followed by Sama-Naguakiri (0.323 mg/kg), while the lowest concentration was seen in Abalama (0.312 mg/kg). The maximum concentration of Zn was observed in Abalama (10.050 mg/kg), followed by Sama-Naguakiri (9.754 mg/kg), while the lowest value was found in Oproama (9.517 mg/kg). The greatest tissue concentration of lead (Pb) was seen in Sama-Naguakiri at 0.144 mg/kg, followed by Oproama at 0.125 mg/kg, while the lowest quantity was found in Abalama at 0.083 mg/kg. The concentration of Cd was found to be higher in Oproama at 0.250 mg/kg, whereas it was lower in Sama-Naguakiri at 0.167 mg/kg. Sama-Naguakiri and Oproama had comparable Cu concentrations (0.0048 mg/kg), which were the greatest, however, the lowest value was seen in Abalama (0.0045 mg/kg). The concentration of Arsenic (As) was found to be consistent at 1.00 mg/kg across all three locations.

**Table 5:** Tissue concentration of heavy metals with reference to water by *P. papillio*.

Locations	Fe	Zn	Pb	Cd	Cu	As
Sama-Naguakiri	0.323	9.754	0.144	0.167	0.0048	1
Oproama	0.326	9.517	0.125	0.250	0.0048	1
Abalama	0.312	10.050	0.083	0.20	0.0045	1

NB: Concentration in (mg kg^−1^).

### 3.7 Biota-sediment accumulation factor (BSAF) of heavy metal in tissue *P. papillio*

Table 6 shows the variations in the tissue concentration of heavy metals in sediment in Sama-Naguakiri, Oproama, and Abalama due to *P. papillio*. The concentrations exhibited significant variations both between heavy metals and across the stations. Among the heavy metals, Zn showed the highest concentration in Abalama (0.486 mg/kg), while the least was recorded in Oproama (0.462 mg/kg). Similarly, the highest Fe concentration was observed in Abalama (0.0072 mg/kg), and the lowest was in Sama-Naguakiri (0.0070 mg/kg). In the case of Pb, the highest values were found in Abalama (0.0014 mg/kg), followed by Oproama (0.0013 mg/kg), with the lowest concentration recorded in Sama-Naguakiri (0.0009 mg/kg). Cd exhibited slightly varied values across the stations, with the highest value (0.004 mg/kg) observed in Oproama and Abalama, while the lowest value was recorded in Sama-Naguakiri (0.003 mg/kg). Copper concentrations also displayed slight differences among the stations. The highest value (0.0015 mg/kg) was recorded in Abalama, followed by Oproama (0.0014 mg/kg), and the lowest concentration was observed in Sama-Naguakiri (0.0013 mg/kg). The sequence of tissue concentration of heavy metals in sediment, from highest to lowest, across each station is as follows: Sama-Naguakiri: Zinc > Arsenic > Iron > Cadmium > Copper > Lead, Oproama: Arsenic > Zinc > Iron > Copper > Lead > Cadmium, Abalama: Arsenic > Zinc > Iron > Copper > Lead > Cadmium.

**Table 6:** The tissue content of heavy metals in *P. papillio* in relation to sediment.

Locations	Fe	Zn	Pb	Cd	Cu	As
Sama-Naguakiri	0.0070	0.476	0.0009	0.003	0.0013	0.5
Oproama	0.0071	0.462	0.0013	0.004	0.0014	1
Abalama	0.0072	0.486	0.0014	0.004	0.0015	1

NB: Concentration in (mg kg^−1^).

## 4 Discussion

### 4.1 Physical and chemical parameters

The temperature ranged between 25.75 ± 0.57 °C in Sama-Naguakiri and 29.50 ± 0.74 °C in Oproama. The recorded temperature range was above the water quality standards (20 °C) recommended by WHO [26]. This outcome aligns with the averages seen in the seasonal variations of Niger Delta waters, as reported by Nafagha-Lawal et al. [28], and a similar trend was observed in the Bonny/New Calabar Estuary [29]. However, Oparaocha et al. [30] reported a maximum water temperature of 28 °C from a different water source in Nigeria, which contrasts with our findings. These surface water temperatures in our study exceeded both the WHO-recommended levels and the optimal temperature range required for some aerobic mesophilic bacteria and fungi. Conversely, Onojake et al. [20] observed a similar temperature reduction, which they attributed to heavy rainfall, given the area's high rainfall amounts and extended rainy season. The change in temperature between the stations may be attributable to several factors, including the velocity of river flow, evident elevation differences, ongoing mixing of organic nutrients, and other anthropogenic activities such as fishing and transportation [[31], [32]]. The elevated water temperature observed in Oproama, measured, could potentially be influenced by various factors, including climatic conditions, geographical features, and groundwater depths. These factors have the potential to impact the physiological and biochemical processes of organisms inhabiting the water sources [33].

The pH of water is a critical factor that is associated with the chemical equilibrium between free hydrogen ions and hydroxide ions in aqueous solutions [34]. The pH values observed in this study were consistent with those reported for the surface water of the Bonny/New Calabar River Estuary in the Niger Delta, Nigeria. These pH values were found to be influenced by the presence of trace metals, as documented by Onojake et al. [29]. Furthermore, the pH values were representative of the tidal brackish water climate, as indicated by previous studies conducted by Onojake et al. [20]. These values are consistent with the pH ranges reported for Isaka-bundu Creek in Rivers state [35] and align with a previous investigation on the physicochemical parameters and heavy metal contents of water in the mangrove swamps of Lagos Lagoon, Nigeria [36]. The measured values were found to be within the acceptable range for water quality (6.5–8.5) as defined by the World Health Organisation [26], indicating that the water is suitable for sustaining marine organisms. The average pH values of 5.68 ± 0.18 observed in Oproama are below the recommended threshold of 6.5 as stated by the WHO [26]. This indicates a somewhat acidic nature, which may give rise to health issues such as acidosis infections [37]. Additionally, it is worth noting that the presence of a low pH in waterbodies might have synergistic effects on the toxicity of heavy metals, as discussed by Adesakin et al. [38]. Research has demonstrated that the pH levels of aquatic plants have a significant impact on the processes of photosynthesis and growth, exerting both direct and indirect effects. According to Davies and Efekemo [18], the presence of elevated pH levels in water leads to an increased production of carbonate and bicarbonate. Conversely, it has been shown that water with a low pH level leads to a reduction in the dissociation of iron phosphate within the solution, as indicated by Lawson [36]. According to Chen et al. [39], it is generally considered that the optimal pH level for potable water is 7, which corresponds to the pH level of pure water. Based on the available evidence, it can be inferred that water sourced from the Oproama, Abalama, and Sama-Naguakiri mangrove swamps in Rivers State, Nigeria, has potential suitability for fish production.

Electricity conductivity is an indication of the electrical properties of water that measures the total dissolved solid (mineral) content of water [[40], [41]]. It also expresses its capacity to conduct electric current. The highest electrical conductivity value was observed at Oproama, this was followed by Abalama and the lowest value was observed in Sama-Naguakiri. Electrical conductivity varied significantly between the studied sites. Electrical conductivity, which measures ion concentration in water, has a significant impact on water flavour and frequently impacts customer preferences for water quality [42]. It is a quick and easy way to determine the total dissolved ions in a water sample and is strongly related to the overall solids content [43].

[44]) found high conductivity values in a number of water systems in the Niger Delta area. Onojake et al. [20] discovered higher and seasonally variable conductivity levels in the Bonny/New Calabar Estuary. The increased conductivity value in Oproama might be ascribed to the substantial input of saltwater. However, given that Oproama routinely gets residential and sewage effluents from a highly populated community, it might possibly be related to the high total dissolved solids load in the water.

The water source in Sama-Naguakiri has a lower conductivity value. This suggests that the water in this area receives a lesser amount of dissolved inorganic compounds in ionized form from its surface catchment regions, as shown by Mezgebe et al. [45]. The observed fluctuations in conductivity might potentially indicate variations in the ionic composition of the water samples, as influenced by the quantity of dissolved particles [46]. The location with the highest recorded biochemical oxygen demand (BOD) value was Oproama. Conversely, the location with the lowest recorded BOD value was Sama-Naguakiri. The recorded biochemical oxygen demand (BOD) values fell within the acceptable range of 10 mg/L as specified by the United States Environmental Protection Agency (USEPA) and the World Health Organisation (WHO). This suggests that the water body in question may be considered relatively safe. The variability seen across the stations might also be ascribed to the presence of a waste heap located at various positions throughout the area. The observed variations in BOD values across the stations might potentially be attributed to factors like as salt levels, temperature, or the degree of decomposition of the deposited chemicals in the vicinity of the stations [[20], [47]]. The BOD range observed in this stream suggests that the water sources have experienced significant pollution from organic substances, including faecal matter, effluents from abattoirs, and incorrect disposal of home waste items. These pollutants have entered the water body through runoff processes. The observed fluctuations in the Biochemical Oxygen Demand (BOD) measurements of the stream can be ascribed to the elevated levels of pollution it encounters, particularly stemming from the discharge of illicitly processed crude oil effluents [48]. The assertion that a decrease in biochemical oxygen demand (BOD) in water may be attributed to the consumption of oxygen by microorganisms, particularly bacteria, for the purpose of oxidising organic substances present in the water, is corroborated by a corresponding study conducted by the World Health Organisation (WHO) in 2008.

Surface water quality assessment relies on various biochemical and chemical constituents, including total dissolved and suspended particles, as well as indicators like dissolved oxygen demand, sulphate, nitrate, chloride, phosphate, and ammonia [31].

These fluctuations in total dissolved solids (TDS) concentrations might signify an increasing level of water quality degradation and pollution in the vicinity of the water body [49]. It's noteworthy that the recorded values fell within the acceptable range of 500 mg/L set by the World Health Organization in 2011. However, when compared to the recommended water quality levels for Eagle Island Creek in the Niger Delta, as proposed by Akankali et al. [50], the results in this research appear relatively lower. This discrepancy may indicate the presence of anthropogenic chemical waste at various geographical sites [51].

The higher TDS content observed in Oproama might be attributed to factors such as increased surface runoff, the presence of surface streams, and a higher discharge of organic debris into the water [18]. Elevated levels of TDS can impact the taste and structural integrity of plumbing systems and related components [52]. Additionally, there is a known correlation between TDS and water conductivity. According to the guidelines established by the United States Environmental Protection Agency (USEPA), water with a dissolved solids concentration exceeding 2000 mg/L is not recommended for use unless other water sources with lower mineral content are available. The salinity of the water, on the other hand, is characterized by a TDS level exceeding 5000 mg/L. This observation suggests that the water within mangrove swamps has the capacity to transition between freshwater and brackish conditions. The observed variations among different stations and months may be linked to the onset of seasonal fluctuations, leading to increased silt input into the creek [50].

This study found that the levels of dissolved oxygen (DO) ranged from 2.92 ± 0.20 mg/L in Oproama to 3.35 ± 0.11 mg/L in Sama-Naguakiri. These findings were in line with similar results reported for polluted water bodies in the Niger Delta [35,36] and all remained within the recommended 6 mg/L threshold for drinking water use [26]. Water can have dissolved oxygen because it can come directly from the air or because autotrophic organisms make it through photosynthesis [53]. According to [37]) and other researchers, various types of organic matter, including human waste, decaying household and sawmill waste, and plant matter close to the sampling stations, may have contributed to the observed differences in DO values.

According to Chapra et al. [54], the extent of oxygen depletion primarily depends on factors such as the amount of waste introduced, stream size and velocity, turbulence, and water temperature. Lawson [36] found higher levels of dissolved oxygen (4.95 ± 3.15 mg/L, 4.97 ± 3.09 mg/L, and 4.91 ± 3.11 mg/L) in the mangrove swamps of Lagos Lagoon. Later studies of the Bonny/New Calabar Estuary also found higher DO levels. It was thought that lower DO levels in water might be due to aquatic plants having less photoperiod and photosynthesis activity [55].

The water temperature may have also influenced the dissolved oxygen levels, as less oxygen dissolves in warm water compared to cold water [31]. Previous research [50] has also shown that runoff from a slaughterhouse and related activities may have also played a part in the lower DO levels in this body of water. This is consistent with Nduka and Orish [56], who found that DO can oxidise both organic and inorganic compounds, potentially reducing their nuisance to consumers.

The study revealed a variation in salinity levels, ranging from 11.44 ± 0.45 ppm in Sama-Naguakiri to 15.28 ± 0.71 ppm in Oproama. It is important to highlight that the salt levels documented in this investigation were found to be greater than the average salinity seen in previous research done on the mangrove swamps of Lagos Lagoon. This earlier study showed a mean salinity of 8.995 ± 0.01 ppm [36].

The increased salt levels seen in this study might likely be ascribed to the close vicinity of the sample sites to the Bonny River, which gets a substantial influx of seawater, hence potentially resulting in tidal impacts [57]. Onojake et al. [58] have documented comparable patterns of elevated salinity levels during dry seasons relative to wet seasons. This observation may be attributed to heightened surface water evaporation caused by strong solar radiation in the dry season, leading to an increase in water salinity [59]. According to Akankali et al. [50], there was an observed elevation in salinity downstream, which was attributed to the closeness of the sampling sites to both the estuary and the sea. The study conducted by Onyema et al. [60] documented the presence of diverse salinity levels across different seasons, which aligns with the results obtained in the present investigation.

It is significant to state that the outcomes acquired in this investigation exhibit disparities in comparison to the discoveries of Akankali et al. [50], whereby they documented the highest and lowest salinity measurements as 4.60 and 1.67, respectively. Nevertheless, the findings are consistent with the fluctuations in salinity that were documented in a preliminary investigation undertaken by Ezeilo and Dune [61]. Magaji and Chup [62] also documented a salinity value of 13,000 ppm obtained from the Gwagwalada region.

### 4.2 The levels of trace metals in water, sediment, and fish

Metals get into the water in a number of ways, including oxidation-reduction reactions, adsorption-desorption reactions, sedimentation, resuspension, and breakdown by living things [63]. Environmentalists generally agree that heavy metals are very bad for the environment because they do not break down and hurt aquatic ecosystems [50].

The sequence of heavy metal distribution in water, fish, and sediment is as follows: Water = Cu > Fe > Zn > Pb > Cd > As; Fish = Zn > Fe > Cu > Pb=As=Cd; Sediment = Fe > Cu > Zn > Pb > Cd > As. Among these metals, Cu, Zn, and Fe displayed the highest values in water, *P. papillio*, and sediment, respectively. The findings in this study align with those reported by Odekina et al. [35] but differ from metal concentrations in various water bodies within and beyond the Niger Delta region of Nigeria. This variation may be attributed to the fact that heavy metal contamination in surface waters and sediment in the Niger Delta is frequently linked to discharges or spills from the oil and gas industry and other human activities in the region [64]. There was no statistically significant difference (P > 0.05) seen in the amounts of As between sediment, fish, and water. Nevertheless, significant disparities in the levels of heavy metal concentrations were observed among various sampling locations, as well as within the fish tissue of *P. papillio*. The observed variations in metal concentrations may be ascribed to the release of untreated or partially treated industrial effluents, as well as residential and household garbage, emanating from the vicinity.

Although the heavy metal distribution in fish tissue showed consistency among stations, the accumulation rates of all metals, except for Arsenic (As), differed between stations. The diversity of aquatic life is influenced by various patterns of chemical distribution in surface waters. Additionally, sediment in the aquatic ecosystem serves as a reservoir for contaminants and has the potential to recontamination the water column. This recontamination process is influenced by factors such as turbulence and bioturbation [31].

The water samples collected from station 3 (Sama-Naguakiri) revealed the greatest amounts of Copper, Zinc, Cadmium (Cd), and Iron. Conversely, station 1 (Oproama) recorded the highest levels of Lead (Pb) in the water. This observation implies that there exists a progression of increased amounts of contaminants moving upstream along the river, with Sama-Naguakiri exhibiting a higher degree of contamination. Omole et al. [65] conducted research that yielded comparable results pertaining to the surface waters of the Atuwara River in western Nigeria. Furthermore, a study conducted by Ololade and Ajayi [66] revealed the presence of high levels of cadmium beyond the suggested thresholds in four significant rivers (Oluwa, Owena, Ogbese, and Ose) located in Ondo State, Nigeria. The water samples had a maximum recorded Iron content of 7.06, although the Arsenic levels remained consistently low at 0.001. In research done by Lawson [36] on the mangrove swamps of Lagos Lagoon, the reported concentrations of Fe ranged from 6.067 ± 1.98 mg/L in Ikoyi to 8.065 ± 1.99 mg/L at UNILAG. These values were found to be lower than the quantities recorded in the present study. According to Franko et al. [67], there has been a documented increase in iron concentrations in surface water throughout tropical aquatic ecosystems during the previous few decades.

The stations had the greatest metal content as shown by the sediment records. The metal content in water was found to be greater for all metals in comparison to the measured concentration in *P. papillio*. According to Islam et al. [68], the findings suggest that there is a gradual and increased accumulation of metals in sediments, which serves as a reservoir for pollutants in aquatic ecosystems. Additionally, there exists a hypothesis suggesting that metals, including cadmium, copper, lead, and zinc, have a pronounced propensity for binding to organic substances within sedimentary environments [69]. Previous studies conducted in other freshwater habitats have shown similar results to our own research, although some have presented conflicting outcomes on the metal composition of freshwater ecosystems in Nigeria [70]. Adesakin et al. [38] discovered that the levels of heavy metals in water were identical, while the levels of metals in sediments in the Ondo Estuary were greater. However, Akankali et al. [50] observed that the levels of heavy metals in the surface waters of the Ala River in Ondo State were the same. A study conducted by Akinbile and Omoniyi [71] documented the presence of decreased metal concentrations in the Ogun and Ogbese rivers located in western Nigeria. In contrast to our study, Ayandirana et al. [72] observed a lower concentration of heavy metals in water samples, while detecting higher levels of metals such as chromium, cadmium, copper, lead, nickel, manganese, and zinc in sediment samples. The capacity of aquatic biodiversity to assimilate and amass contaminants within freshwater environments is well-documented.

### 4.3 Variation in heavy metals concentration across the station

In comparison to Oproama and Abalama, Sama-Naguakiri had elevated concentrations of Fe, Zn, Pb, Cd, and Cu. Oproama had quantities of intermediate magnitude, whereas Abalama consistently showed the lowest concentrations of these metals among the three studied places. Statistically significant variations (P < 0.05) were identified in the concentrations of each heavy metal across the three locations. The observed sequence of metal concentrations may be described as follows: Fe exhibited the highest concentration, followed by Cu, Zn, Pb, Cd and As. Generally, Sama-Naguakiri exhibited slightly higher metal concentrations compared to Oproama and Abalama, except for As, which had similar concentrations across all stations. Vincent-Akpu et al. [73] conducted a study at Elechi Creek, Port Harcourt, Rivers State, Nigeria, where they noted the same trend with trace metals found in water, fish, and sediments. Therefore, the observed low levels of Arsenic (As) in this study may be attributed to the inherent characteristic of marine settings, which often exhibit low concentrations of As [74]. It is more commonly found in freshwater systems and in soils, where it can enter the water supply through agricultural and industrial activities.

In marine ecosystems, the concentration of arsenic is often low as a result of the dilution effect caused by the substantial water volume, as well as the inherent capacity of some marine species to eliminate arsenic from the water through natural mechanisms [75]. However, arsenic can enter marine environments through the discharge of waste, and it can also be present due to natural geological processes [76]. Cu, Zn, Pb, and Fe are all elements that are abundant in marine environments. These harmful components might have gotten into the stations from a number of sources, including rainwater runoff, and natural weathering of rocks and minerals [77]. In polluted marine environments, these elements can accumulate in sediment, fish, and water due to their ability to bind to organic matter and particulates in the water column [[78], [79]]. This can result in elevated concentrations of these elements in these matrices, potentially causing detrimental effects on the well-being of marine organisms and ecosystems [76].

### 4.4 Bio- water accumulation factor (BAF) of heavy metals in water in tissue *P. papillio*

In freshwater ecosystems, aquatic biodiversity has the capacity to take in and accumulate pollutants [80]. Many biological agents have the potential to amplify pollutants in their tissues above levels seen in the environment [31]. The greatest Fe content in *P. papillio* tissue by water is between 0.312 and 0.326 mg/kg. Because the fish has absorbed this quantity of iron from the water, this is an indication of the degree of iron pollution in the water [81]. Because higher levels of iron pollution can be detrimental to people and other species, this information can be used to estimate the risk of contamination to humans and other organisms in the region [82]. In this investigation, Zn value was higher than one (BAF >1), indicating effective contaminant absorption.

A BAF value greater than one suggests that a pollutant is efficiently absorbed by an organism [83]. This indicates that the pollutant can accumulate in larger quantities than in the environment, which might indicate possible health problems [84]. BAF levels must be measured in order to determine the possible effects of pollutants on the environment and human health. The mean amounts of Pb and Cd in the tissue of the bioaccumulated fish species (*P. papillio*) vary between 0.083 and 0.144 mg/kg for Pb, 0.312 and 0.326 mg/kg for iron (Fe), and 0.167 and 0.25 mg/kg for Cd. Furthermore, the Bioaccumulation Factor (BAF) for Pb, Cd, and Fe was less than one, showing that these metals were eliminated from fish tissue [85].

This means that the mean concentrations of arsenic (As) in the tissue of the fish species (*P. papillio*) that was bioaccumulated from the water was equal to one, indicating that these metals are accumulating in the fish tissue [86]. This is an indication of potential health risks associated with the consumption of this fish species, as arsenic can be toxic to humans in high concentrations [87]. It is important to monitor the concentrations of arsenic in fish tissue to assess the potential health risks associated with the consumption of this species. From the results, a distinctive variance was observed in tissue concentration of the heavy metals in *P. papillio* across the stations.

This indicates that the level of iron (Fe) in fish tissue (*P. papillio*) bioaccumulated from the water was greatest in Oproama, followed by Sama-Naguakiri, and lowest in Abalama. This implies that the quantities of iron in the water at these various places fluctuate, most likely due to varying degrees of pollution in the water. The differences might be caused by a range of variables, such as industrial operations or effluent from artisanal crude oil refining waste runoff, which can contribute to higher amounts of heavy metals in water bodies [88]. It's also worth noting that heavy metal pollution can harm the health and survival of many fish species [89].

The observed levels of zinc (Zn) in these specific locations may stem from various sources, such as point-source pollution from nearby industries or diffuse pollution sources like agricultural runoff [90]. Furthermore, specific marine organisms like *P. papillio*, mussels, and clams are capable of accumulating substantial amounts of zinc in their tissues, contributing to elevated zinc concentrations in both these organisms and sediments. Variations in zinc concentrations among different locations can be linked to local geological characteristics and water flow patterns, influencing the distribution of pollutants. It's important to note that, similar to other heavy metals, high levels of zinc can be toxic to marine organisms and humans, underscoring the need for monitoring zinc levels to protect ecosystems and human health [91].

Lead (Pb) exhibited higher tissue concentrations in Sama-Naguakiri and Oproama, while Abalama had the lowest levels. This suggests a localized source of lead pollution in the former two areas, potentially originating from industrial activities such as illegal crude oil refining or other human practices [[24], [27]]. Lead can bioaccumulate marine organisms, posing risks to both organism health and humans who consume them [12]. Identifying and addressing the source of lead pollution is crucial to mitigate further contamination. Cadmium (Cd) showed higher tissue concentrations in Oproama and lower levels in Sama-Naguakiri, implying localized cadmium pollution in Oproama. Similar to lead, this could stem from industrial facilities or other human activities releasing cadmium into the environment [76]. Cadmium, being a toxic heavy metal, can accumulate in fish and shellfish tissues, posing risks to both marine organisms and humans [92]. Investigating the pollution source and implementing measures to prevent and reduce contamination is essential.

Copper concentrations were similar in Sama-Naguakiri and Oproama but lower in Abalama, suggesting a shared pollution source in the former two areas. However, geological differences or variations in water flow might contribute to the lower copper levels in Abalama. Copper is essential for marine organisms and humans, necessitating ongoing monitoring to ensure safe levels for both ecosystems and the health of humans [93].

Arsenic (As) concentrations in *P. papillio* were similar across the studied stations, indicating consistent levels of arsenic pollution in the water. The value of 1.00 mg/kg suggests comparable concentrations of arsenic across locations. Monitoring arsenic levels remains crucial to assess its impact on the ecosystem and human health.

### 4.5 Bio-sediment accumulation factor (BSAF) of heavy metal in tissue *P. papillio*

The results reveal that the difference in heavy metal tissue concentrations in *P. papillio* from sediment varied considerably between heavy metals and stations (Sama-Naguakiri, Oproama, and Abalama). The following is the order of tissue concentrations of heavy metals in sediment from highest to lowest throughout each station: Oproama: Arsenic > Zinc > Iron > Copper > Lead > Cadmium, Sama-Naguakiri: Arsenic > Zinc > Iron > Copper > Lead > Cadmium, Abalama: Arsenic > Zinc > Iron > Copper > Lead > Cadmium.

The increased zinc content in Abalama is most likely attributable to the fact that it is a shallow, slow-moving body of water with more silt and organic debris, which can function as a sink for heavy metals [94]. The lower zinc content in Oproama is likely attributable to the fact that it is a deeper, faster-moving body of water with less silt and organic matter, reducing the number of heavy metals that might be collected [95]. The presence of organic debris, which might function as a sink for the metal, is believed to be responsible for the highest concentration of iron in Abalama. The lower concentration of iron in Sama-Naguakiri is likely due to the fact that it is a deeper, faster-moving body of water, which reduces the amount of iron that can accumulate [96]. The highest values of Pb in Abalama and Oproama soil samples could be a result of industrial activities in the area as well as agricultural activities such as the use of lead-based fertilisers, and pesticides, The lowest value found in Sama-Naguakiri might be attributable to the soil being less exposed to anthropogenic lead sources [[97], [98]].

Cd contents in the sediments of the analysed locations were found to be less than the permitted limit of 0.1 mg/kg imposed by the Nigerian Environmental Standards and Regulations Enforcement Agency (NESREA). The findings also indicate that the soils in the assessed areas are not polluted with Cd and are appropriate for agricultural use. As a result, more research should be carried out to determine the sources of Cd in the soils of the analysed locations, as well as to assess the possible ecological and health hazards of Cd pollution [[99], [100]]. The greater copper content in Abalama is most likely owing to the presence of organic debris, which can function as a metal sink [[101], [102]]. The lower copper concentrations in Oproama and Sama-Naguakiri are most likely owing to deeper, faster-moving bodies of water, which limit the amount of copper that may be collected [95].

The synergistic interaction between the heavy metal content in *P. papillio* tissue and sediment is a crucial aspect that requires thorough exploration. The complex relationship between these environmental compartments significantly impacts the bioavailability, accumulation, and potential effects of heavy metals on aquatic organisms and the ecosystem [63].

Metals such as copper (Cu), zinc (Zn), lead (Pb), cadmium (Cd), and iron (Fe) follow distinct pathways in aquatic environments, moving through water, sediment, and organisms [50]. Understanding the dynamics of this metal transfer is essential for unraveling the complexities associated with their ecological implications.

In terms of heavy metal distribution, sediment acts as a vital reservoir, accumulating contaminants from sources like atmospheric deposition, surface water runoff, and industrial discharges [[66], [103]]. According to Chris et al. [18], the physical and chemical properties of sediment, including composition, texture, and redox conditions, play crucial roles in metal sorption and retention.

*P. papillio*, as a representative aquatic organism, interacts closely with sediment through feeding, respiration, and habitat use. The bioaccumulation of heavy metals in fish tissues reflects the amalgamation of metals from water and sediment [[35], [104]]. Sediments, as a primary source, release metals into the water column, which are then absorbed by aquatic organisms like *P. papillio*.

The observed consistencies or discrepancies in heavy metal concentrations between *P. papillio* tissues and sediment at different sampling stations highlight the localized nature of contamination sources. According to Abdollahi et al. (2019), this could be due to sediment characteristics, proximity to pollutant sources, and local human activities.Variations in metal accumulation rates, excluding arsenic (As), among sampling stations indicate complex chemical dynamics in surface waters. Sediment, as a primary pollutant repository, plays a key role in regulating heavy metal bioavailability. Davies and Ekperusi [[31], [105]] stated that turbulence and bioturbation affect the recontamination of the water column, contributing to metal cycling in aquatic ecosystems.

Further research into the synergistic relationship could focus on understanding the mechanisms of metal uptake by *P. papillio* from sediment, considering sediment characteristics, seasonal changes, and specific assimilation pathways. Exploring the potential impacts of sediment-related heavy metals on the health and behavior of *P. papillio* could offer insights into the broader ecological consequences of metal contamination in aquatic ecosystems.

## 5 Conclusions

This study aimed to assess the impact of illicit and small-scale crude oil refining and industrial activities on water quality and food safety in the Niger Delta region. The identification of hazardous metals in water, sediment, and mudskippers raised concerns about their potential negative effects on human health and the ecosystem. The study found variations in metal pollution levels among different regions due to distinct sources of contamination. The analysis also revealed that fish tissues, which are consumable constituents, had heightened concentrations of heavy metals. The different amounts of metals found in the tissues suggest that the tendency for heavy metals to build up varies between sampling sites and may be more focused on certain metals. This study's major outcome underscores the health hazards associated with consuming a specific fish species in different geographical locations. Future prospects entail further examination and surveillance to address specific risks in these areas, emphasising the need for targeted interventions and regulatory measures.

## Funding

No funding was obtained for this study.

## Data availability statement

The datasets generated during and/or analysed during the current study are available from the corresponding author on reasonable request.

### Consent to participate

Not applicable.

## CRediT authorship contribution statement

**Davies Ibienebo Chris:** Writing – review & editing, Writing – original draft, Funding acquisition, Formal analysis, Data curation, Conceptualization. **Nwosu Obiageli Juliana:** Resources, Project administration, Methodology, Investigation, Funding acquisition, Formal analysis, Data curation, Conceptualization. **Okechukwu Kenneth Wokeh:** Visualization, Validation, Supervision, Software, Resources, Project administration. **Azra Mohamad Nor:** Writing – review & editing, Writing – original draft. **Fathurrahman Lananan:** Visualization, Validation. **Lee Seong Wei:** Writing – review & editing, Writing – original draft.

## Declaration of competing interest

The authors declare the following financial interests/personal relationships which may be considered as potential competing interests:

Lee Seong Wei is an Associate Editor of Heliyon If there are other authors, they declare that they have no known competing financial interests or personal relationships that could have appeared to influence the work reported in this paper.
